# The Hospital of St. John and St. Elizabeth

**Published:** 1901-07-20

**Authors:** 


### The Institutional Workshop.

### THE HOSPITAL OF ST. JOHN AND ST. ELIZABETH.

The Lord Mayor of London opened the new Hospital of St. John and St. Elizabeth, at St. John's Wood, on Monday afternoon, in the presence of a large company; among the many notable Roman Catholics present being the president of the hospital (the Duke of Norfolk), the chairman of the Board of Management (Lord Clifford of Chudleigh), the Dowager Duchess of Newcastle, Lady Clifford of Chudleigh, the Marquis of Ripon, Lord and Lady Denbigh, Lord Ashburnham, Lord and Lady Edmund Talbot, Lord and Lady Brampton, Lord and Lady Gainsborough, Lord Abingdon, and Lord and Lady Arundell of Wardover. The hospital was originally founded in Great Ormond Street in 1856, by Cardinal Wiseman and the late Earl of Gainsborough. The object was to receive chiefly two classes of patients—those who suffered from incurable disease, especially when near death, and those whose maladies, though not necessarily incurable, required long-continued treatment—and two houses having been purchased for the purpose, accommodation was provided for 50 or 60 patients. The Crimean War had just ended, and a band of sisters of mercy from Bermondsey, who had been nursing our soldiers at the seat of war, were asked to undertake the nursing at the hospital, which was dedicated to St. Elizabeth of Hungary. It subsequently became the Hospital of St. John and St. Elizabeth, when the late Sir George Bowyer reintroduced the Order of Malta, Knights Hospitallers of St. John of Jerusalem, into England, and built a church on the ground occupied by one of the houses, the other being so enlarged as to admit the same number of patients as the two houses had received. A convent for the sisters was included in the plan, and they have ever since worn the Cross of Malta on their habits. In 1896 the building in Great Ormond Street, then used as the hospital, was condemned as unfit for use, and it became necessary to consider the question of rebuilding at once. Ultimately, owing partly to the imperative need of extending the Hospital for Sick Children, it was decided to remove to St. John's Wood.

The hospital, which has now been completed, is entirely new; but the church, with its dome, has been transferred from the place where it stood, stone by stone. Its beauty is undeniable, and the authorities must be congratulated upon the manner in which the work of removal has been accomplished. Near the roof are small galleries, almost hidden from sight so as not to interfere with the symmetry of the whole, in which patients from the wards, not well enough to come downstairs yet sufficiently convalescent to be able to enjoy the service, can hear without being seen. The hospital itself is much more commodious than the old one, and will hold 110 beds. The wards are large, well ventilated, and lighted by electricity. Every modern appliance has been placed in them, and the furniture is admirable —that in one wing being the gift of a Roman Catholic priest, whilst Lord and Lady Clifford, of Chudleigh, gave many of the articles of domestic and culinary use. During the past year three beds have been founded, (1) "The Angel Guardians," (2) "In Memoriam of Captain Haydock," (3) the "May Purcell" bed, and a fourth has been promised and partly paid for. An interesting fact is that a cot in the Children's Ward has been generously presented by the workmen on the new building. There is a well-appointed operating room, the lavatory accommodation is excellent, and the lifts will be a great convenience. The situation of the hospital is singularly good. From the roof-walk, which eventually will possess awnings, and is intended for the use of the patients who are well enough to be in the open air, a splendid view of the Surrey hills on one side, and Highgate and Harrow heights on the other, can be obtained.

Before the Lord Mayor declared the building open, Lord Clifford of Chudleigh delivered in one of the vacant wards, a short address, in the course of which he explained the circumstances which had led to the removal. As to the cost, he stated that the erection of the new hospital had reduced the invested capital from £23,000 to £9,000, and that the sum of £4,000 was still owing to the builders and architect. He also explained that the Board of Management were forced by the Charity Commissioners to replace the amount drawn from their invested capital at the rate of £500 a year. To keep things going with the present number of patients, the hospital required an income of about £2,000 a year, and to fill it another £1,000. It was proposed, by an arrangement with the Soldiers' and Sailors' Families' Association, to take into the vacant wards some of the men who had been severely wounded in South Africa.

At the conclusion of the address, the majority of the guests, headed by the Lord Mayor, with whom were the Lady Mayoress and the Sheriffs, perambulated the wards, and visited every portion of the building. The chief magistrate, in discharging the formal ceremony, dwelt on the fact that sufferers of all shades of religious opinion are admitted to the hospital. The Duke of Norfolk in moving a vote of thanks to the Lord Mayor for his attendance, intimated that the patients would, as formerly, be under the care of the Prioress and Sisters of Mercy of the Convent of St. John. The band of the Grenadier Guards, who played at intervals during the afternoon, then struck up "God Save the King," and the proceedings terminated.

